# Burnout, Identity Loss and Institutional Gaps: A Qualitative Examination of Sport Discontinuation Among NCAA Division III Athletes

**DOI:** 10.3390/sports13040116

**Published:** 2025-04-11

**Authors:** James Stavitz, Ryan Porcelli, Jennifer Block-Lerner, Donald R. Marks, Hallie Katzman

**Affiliations:** 1 Department of Athletic Training Education, Kean University, 1000 Morris Avenue, Union, NJ 07083, USA; 2 Department of Advanced Studies in Psychology, Kean University, 1000 Morris Avenue, Union, NJ 07083, USA

**Keywords:** mental health, burnout, identity loss, NCAA Division III, sport discontinuation, athlete well-being

## Abstract

Mental health challenges significantly impact NCAA Division III student athletes, often leading them to discontinue their sport. Unlike Division I athletes, they have limited institutional support, making it harder to balance academic and athletic demands. This qualitative study examines the mental health barriers influencing 21 former Division III athletes’ decisions to withdraw. Key themes include stress, burnout, identity loss, inadequate institutional support and external pressures like academics and finances. The findings highlight the need for better mental health resources, reduced stigma, and stronger institutional support. Addressing these challenges can improve athlete well-being and retention in Division III programs.

## 1. Introduction

Mental health challenges are increasingly recognized as significant factors that lead NCAA Division III student-athletes to stop participating in sports [1]. Unlike their Division I counterparts, Division III athletes often lack access to adequate mental health resources, which makes it harder to manage the stress of balancing academic responsibilities with athletic demands [2]. Research shows that student-athletes face a higher risk for depression, anxiety, emotional exhaustion and identity disturbance due to personal expectations and external pressures from coaches, peers and academic commitments [3].

There are over 440 NCAA Division III institutions supporting nearly 190,000 student-athletes across the United States. This level of competition emphasizes balance between academics and athletics, encouraging student-athletes to focus primarily on their education [4,5,6]. Unlike Divisions I and II, Division III athletes do not receive athletic scholarships and must prioritize academics while managing the demands of athletic participation with fewer resources and less institutional support [1,7]. Although most mental health studies focus on Division I athletes, Division III student-athletes face distinct challenges, including limited support, financial stress, coaching changes and the ongoing struggle to balance academics and athletics [8,9]. While these challenges are increasingly acknowledged, institutional responses remain inconsistent, and access to athlete-specific mental health services is often limited or generalized. Studies show that stigma, particularly among male athletes, continues to deter help-seeking behaviors, while time conflicts and a lack of trained sport-specific providers further reduce access to care [10,11].

According to Stokowski et al. [7], Division III athletes may experience distress levels comparable to their Division I peers, yet mental health initiatives at Division III institutions are often underdeveloped or inconsistently applied. These gaps leave athletes vulnerable, especially when academic and athletic demands intensify. Recent data from the NCAA Student-Athlete Well-Being Study (2020) found that 30% of Division III athletes reported feeling overwhelmed, and 25% reported feeling mentally exhausted either constantly or most of the time [7].

Despite growing awareness, few studies directly explore the lived experiences of former Division III athletes who voluntarily discontinued sport due to mental health-related factors. Much of the existing literature focuses on prevalence data or inter-division comparisons rather than qualitative insights into athlete decision making, identity disruption and emotional burnout [10,11].

The goal of this study is to examine the mental health barriers that influence former NCAA Division III student-athletes to leave their sports. This study explores three key questions: What are the main mental health challenges former Division III athletes experienced during their participation? How did these athletes view the availability and adequacy of institutional mental health resources? What external factors, such as family and social expectations, contributed to their decision to stop participating? This research seeks to identify critical mental health barriers to help develop strategies that support the well-being and retention of student-athletes in NCAA Division III.

## 2. Methods

### 2.1. Study Design

The research team consists of a certified athletic trainer (AT) with a Doctor of Philosophy (PhD) in Health Science and 12 years of licensure and professional experience, a first-year Master of Science in Athletic Training (MSAT) student, two clinical psychologists with Doctor of Psychology (PsyD) and/or PhD degrees, and a doctoral candidate in school and clinical psychology who is also a former Division III athlete. This team integrates expertise in sports medicine, behavioral health and psychological research to examine the mental health challenges that led former NCAA Division III athletes to discontinue their sports participation.

This study employs a qualitative transcendental phenomenological design, selected for its emphasis on exploring the lived experiences of participants and uncovering the essence of a phenomenon as experienced by those who lived it [12,13]. This approach was appropriate because our primary goal was to understand how former student-athletes personally experienced mental health struggles, institutional support gaps, and external pressures, not merely how they are described or explained.

Guided by Moustakas’ 1994 transcendental phenomenology, this study follows a structured approach to epoche (bracketing), phenomenological reduction and imaginative variation to uncover the essence of the participants’ experiences [14]. Bracketing involved the research team’s deliberate effort to identify and suspend their preconceptions about sport discontinuation, burnout, and institutional mental health systems. To achieve this, each member maintained reflexive journals throughout the study and engaged in ongoing team discussions to examine how their backgrounds (e.g., clinical, athletic and psychological) could shape interpretation. Specific preconceptions bracketed included beliefs about the centrality of athletic identity and the assumption that institutional support was inherently insufficient [14].

Phenomenological reduction was operationalized by prioritizing participants’ first-person narratives and reducing data to thematic descriptions rooted directly in their lived accounts, while imaginative variation allowed the team to explore different structural conditions and contexts under which these experiences may manifest [12,13,14].

The semi-structured interview format facilitated open-ended discussions that allowed participants to reflect on their mental health struggles, available support systems and the external pressures that contributed to their decision to stop participating in athletics. For those who opted to complete the survey version of the interview protocol (*n* = 3), we made sure that the questions mirrored the in-depth prompts used in the interviews. Though these participants did not engage in real-time conversation, they provided narrative responses that allowed for a similar level of phenomenological reconstruction. Their data were analyzed using the same inductive and iterative processes.

### 2.2. Participants

This study included 21 former NCAA Division III student-athletes, 13 males and 8 females, who voluntarily discontinued their sport. Purposive sampling ensured diverse perspectives across team and individual sports, academic years and fields of study [15]. Participants were eligible if they were at least 18 years old and had completed at least two competitive seasons in a Division III program, unless they withdrew early due to a significant mental health crisis. They also had to have formally left their sport between one and five years before recruitment. Participants were required to self-report significant psychological distress such as anxiety, depression or burnout as a contributing factor in their decision to stop participating. Those who did not meet these criteria were excluded.

Recruitment to this study used multiple outreach strategies to make sure there was a diverse participant pool [16]. University athletic departments were asked to share study details with eligible alumni and a letter of solicitation was sent to potential participants with a link to a Qualtrics prescreening survey. Recruitment messages were also posted on social media platforms, including LinkedIn, Facebook, Instagram and Snapchat, to reach former student-athletes and NCAA Division III alumni. Personalized emails were sent to athletic program directors and coaches to request their assistance in sharing the study information. Of the 2000 former Division III athletes contacted through the various recruitment strategies, 33 began the inclusion survey, 25 completed it, and 21 provided an email for follow-up to schedule an interview.

### 2.3. Interview Protocol

**Figure 1:**
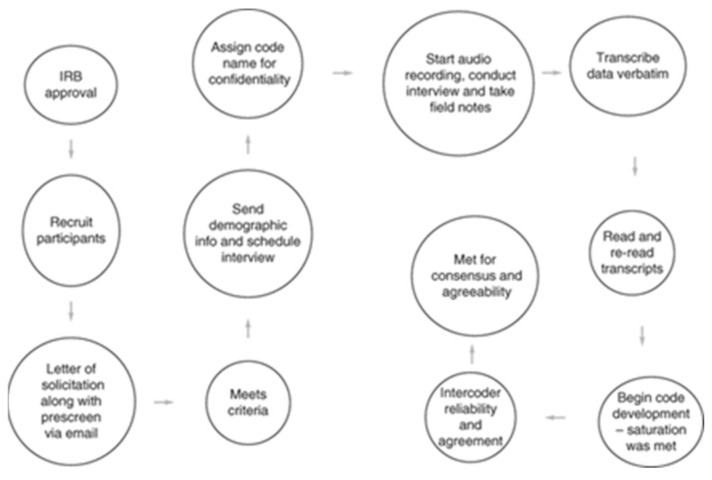
Interview process.

Data were collected through semi-structured, in-depth interviews to examine the mental health challenges and barriers that contributed to the decision of former NCAA Division III athletes to discontinue their sports participation (Figure 1) [17]. The interview protocol was developed based on relevant literature and consisted of open-ended questions addressing personal mental health experiences, perceived support systems, and external influences such as family, academic and social pressures (Table 1) [18,19]. To enhance the quality and relevance of the guide, it was reviewed by a multidisciplinary team including experts in athletic training, sport psychology and qualitative research. Additionally, the protocol was pilot tested with two individuals (one former Division III athlete and one licensed mental health professional) to evaluate clarity, flow and emotional sensitivity. Minor revisions were made based on their feedback. Participants were encouraged to provide detailed narratives and specific examples of the challenges they encountered. Follow-up questions were used to explore key themes further and clarify responses. A codebook was developed iteratively, incorporating emergent themes identified throughout the interview process [20].

### 2.4. Procedures

**Figure 2:**
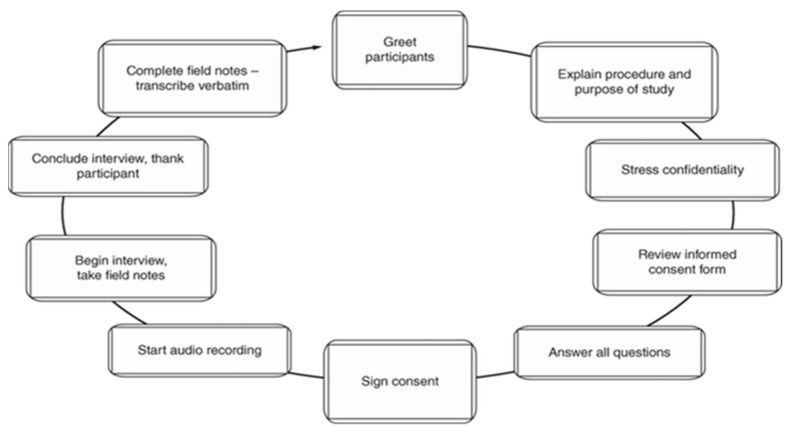
Data collection process.

The research team contacted all potential participants who met the inclusion criteria via email. They provided detailed information about the study’s purpose, procedures and voluntary nature of participation. Each participant received an informed consent form outlining their rights, confidentiality measures and the ability to withdraw at any time. Upon obtaining consent, interviews were scheduled and conducted through a secure video conferencing platform or via telephone, based on participant preference. Each interview lasted approximately 60 to 90 min, allowing participants sufficient time to share their experiences in depth (Figure 2).

All interviews were audio recorded with participant permission and transcribed verbatim using transcription software. To ensure transcription accuracy, the primary investigator reviewed each transcript by cross-referencing it with the original audio recordings. Participants were invited to review their transcripts and clarify their responses if they wished, offering an opportunity for input without requiring participation. This process was entirely optional and was not implemented as a formal validation strategy. For participants who were unable to schedule an interview, an alternative option was offered, allowing them to respond to the interview questions in writing through a Qualtrics survey [21].

Data collection was conducted uniformly across all participants to maintain methodological consistency. The process continued until data saturation was reached, meaning no new themes emerged from additional interviews, indicating sufficient depth of data [20]. All recorded and transcribed data were stored on a password-protected system that was only accessible to authorized research team members.

### 2.5. Data Analysis

**Figure 3:**
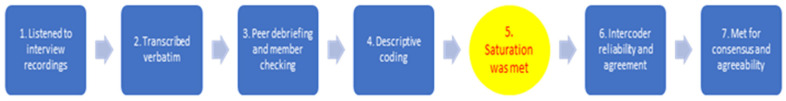
Data analysis process.

Data analysis (Figure 3) involved a thematic process grounded in transcendental phenomenology, used to explore the lived experiences of former NCAA Division III athletes and the mental health barriers that contributed to their decision to discontinue athletic participation [22].

The research team manually transcribed the interviews and engaged in an iterative, reflective analysis of the transcripts. Each member independently read the initial transcripts and documented experiential descriptions and meaning units relevant to the research questions. These units were clustered into preliminary themes and refined collaboratively through group dialogue. The team engaged in reflective discussions to explore the structures underlying each participant’s experience and to distill invariant constituents of meaning [23].

Rather than assessing inter-coder reliability, the team emphasized interpretive dialogue and consensus building to ensure coherence of meaning across analyses. Thematic development was recursive and grounded in close reading and re-reading of participant narratives. We moved away from traditional notions of accuracy and validity, instead establishing trustworthiness through methodological transparency, reflexivity and collective interpretation [24].

While we initially quantified the number of excerpts per code, this approach was removed to better reflect a phenomenological emphasis on the depth and significance of individual experiences rather than their frequency. We have replaced references to “codes” with subordinate themes, clustered under five major thematic domains. This shift allows us to highlight important yet less common experiential accounts that provide valuable insights into the phenomenon.

An external consultation was conducted to gather interpretive feedback from colleagues experienced in qualitative methods. Although participants were still given the opportunity to review their responses upon request, this process was optional and limited in scope. Member checking was not implemented as a formal validation step, in alignment with critiques by Smith and McGannon (2018), who argue that it can misrepresent the fluidity of meaning making and potentially obscure the relational dynamics between interviewer and participant [25]. Instead, we relied on collective reflexivity and sustained engagement with the data to support interpretive depth.

Field notes were recorded during and after interviews to capture contextual information such as tone, pauses, emotional responses and non-verbal cues (when observable via video) [22,23]. These notes were later used to support interpretive analysis, enrich meaning unit development and guide team reflexivity. For written responses, field notes captured the research team’s reflections on content clarity and interpretive tone [22,24].

Methodological coherence was maintained through the use of reflective journaling, regular analytic discussions and the documentation of analytic decisions through an audit trail [26,27,28]. Reflexivity was central to this process and allowed the team to continuously examine how their own professional and experiential backgrounds influenced data interpretation [29,30].

### 2.6. Ethical Considerations

This study obtained IRB exemption from the Kean University Institutional Review Board: IRB-FY2025-31. All participants gave consent and were informed of their right to withdraw. Identification codes were assigned to protect confidentiality. Personal information was removed. All data were stored on a password-protected server. Participants, if they chose to, had the opportunity to review and clarify their transcripts through member checking. Due to the sensitive nature of the topic, they could skip any question. Mental health resources were available if needed. This study followed the guidelines of the Belmont Report and upheld the principles of respect, beneficence and justice [31].

## 3. Results

This study included 21 former NCAA Division III student-athletes who voluntarily discontinued their sport. Participants represented various team and individual sports, with 13 males and 8 females; the mean and median ages at departure were 20.19 and 20 years, respectively. Most athletes left during their sophomore or junior year, often due to injury, mid-season withdrawal or academic and athletic pressures. Table 2, Table 3, Table 4, Table 5, Table 6 and Table 7 provide detailed breakdowns of sports, academic majors, departure timing and reasons for discontinuation, with mental health struggles and burnout being the most common factors.

### 3.1. Themes

Five major themes derived from participants’ experiences, including internal mental health challenges, perceptions of institutional support, and external influences such as family and social expectations (Table 8). These themes help explain the various challenges Division III student-athletes face and show how mental health issues connect to their academic and athletic responsibilities. The findings aim to inform better strategies to support the mental well-being and retention of student-athletes in NCAA Division III programs.

### 3.2. Theme 1: Emotional and Mental Health Challenges in Division III Athletics

This theme captures the profound emotional and mental health challenges faced by NCAA Division III student-athletes. Participants highlighted stress, burnout, identity loss and the emotional toll of leaving their sport, all of which significantly influenced their well-being and decision to discontinue participation. Table 9 presents a summary of the codes, participant involvement and transcript excerpts that contributed to this theme.

#### 3.2.1. Code 1: Stress and Burnout

Eight participants identified stress and burnout as central to their experiences, contributing to fifteen excerpts. Participants frequently described the immense difficulty of managing the competing demands of athletic, academic and personal responsibilities, often leading to physical and emotional exhaustion.

“Yeah, being a student-athlete was kinda intense. It was cool at first—like, having a team and all that. But juggling practices, games, and homework was no joke. It felt like I never had time to just chill or hang out with friends”, shared P3.

P12 echoed this sentiment, stating, “Balancing sports with school and everything else was honestly rough. I always felt like I was falling behind somewhere—either my grades or just having a life outside of sports. It was like I was stuck in this endless loop”.

Several participants highlighted how the relentless schedule wore them down. P8 recalled, “Waking up at 4:00 AM and going to bed after midnight just to fit everything in—it wore me out completely. By the end of the season, I didn’t even recognize myself. I was so drained”.

Others described the long-term impact of this stress. P11 explained, “I felt like I was constantly trying to keep up with everything, and even when I succeeded, it didn’t feel like enough. It was exhausting and made me question why I was doing it in the first place”.

For some, the stress extended beyond athletics. P15 noted, “The pressure to perform on the field, keep up in class, and still have a social life—it just got to be too much. I was always tired, always stressed, and it just wasn’t worth it anymore”.

#### 3.2.2. Code 2: Loss of Enjoyment and Purpose

Six participants shared that the pressures of collegiate athletics eroded their initial enjoyment and sense of purpose, contributing to ten excerpts. Many reflected on how their love for their sport diminished over time.

“I dreaded going to practice. It stopped being fun and just felt like another thing I had to do”, said P7. Similarly, P14 described, “The pressure to perform and the constant time commitment made it hard to enjoy the sport. I wanted to quit because I wasn’t happy anymore”.

Other participants noted that external factors, such as coaching dynamics or institutional expectations, played a role. P17 shared, “I loved football in high school, but by the time I got to college, it felt like a chore. The coaches only cared about winning, not us as people. It was hard to stay passionate when you felt like just another number”.

For P9, the demands of the sport overshadowed its positives: “I thought I loved it, but the reality of always being on and never having time for myself made me realize I was losing that love. It became something I resented”.

#### 3.2.3. Code 3: Depression and Anxiety

Five participants directly referenced struggles with depression and anxiety, with these issues contributing to eight excerpts. These feelings were often tied to the relentless pressure and perceived lack of support.

P8 reflected, “I was very depressed and sad and would cry most nights while playing. It felt like no one understood how much I was struggling”.

P11 described how anxiety impacted their daily life: “I was stressed out all the time. Practices and games made me nervous, and messing up would weigh on me for days. It felt like I could never escape the pressure, even when I was off the field”.

For others, anxiety and depression were amplified by their athletic identity. P6 shared, “It felt like everything was riding on my performance. If I wasn’t playing well, I wasn’t good enough—not just as an athlete, but as a person”.

Institutional support also played a significant role in participants’ mental health. P9 explained, “Football used to be my escape, but in college, it became my biggest source of stress. There wasn’t anyone to turn to when I was struggling, and that just made it worse”.

#### 3.2.4. Code 4: Emotional Toll from Leaving the Sport

Seven participants highlighted the emotional toll of leaving their sport, with 12 excerpts addressing feelings of relief, regret or profound sadness.

P15 expressed mixed emotions, stating, “Honestly, after I decided to quit, I felt kinda relieved, like a huge weight was off my shoulders. I missed some parts of it, but mostly it just felt good to have my time back and not be constantly stressed”.

Others described a deeper sense of loss. P19 shared, “I still regret stepping away. It was incredibly hard to leave the sport, and I miss it every day. It feels like I lost a part of myself”.

For P6, leaving the sport also meant leaving behind a core part of their identity: “Losing sports created a sense of loss for me. It was all I had ever done, and without it, I didn’t know who I was anymore. It felt like starting over”.

P10 reflected on the complexity of the decision: “I knew it was the right choice for my mental health, but that didn’t make it any easier. Walking away from something you’ve poured your life into is never simple”.

#### 3.2.5. Code 5: Burnout and Reflection on Personal Goals

Burnout emerged as a critical factor, mentioned by six participants in nine excerpts. Participants described reaching a breaking point where the demands of their sport no longer aligned with their personal goals or values.

“I realized I couldn’t keep putting everything into the sport and ignoring my academics. It just wasn’t sustainable”, explained P5. This sentiment was echoed by P10, who stated, “I felt like I had no time to myself. I couldn’t balance everything, and it wasn’t fair to my team to keep pretending I could. It got to a point where I had to step back and think about what mattered most”.

Others used their burnout as an opportunity for reflection, reassessing the priorities in their lives. P14 shared, “I loved the sport, but I had to be honest with myself. Was I doing this because I wanted to or because I felt like I had to? That question made me realize it was time to move on”.

For some, burnout was coupled with frustration about their perceived lack of progress or recognition in athletics. P8 reflected, “I kept pushing myself, thinking it would pay off, but it just left me feeling empty. I wasn’t growing as a person or as a student, and I had to ask myself if it was worth it”.

Several participants tied burnout to a desire to focus on long-term goals outside of athletics. P19 explained, “I loved playing, but it felt like I was sacrificing everything else—school, friends, my mental health. I wanted to build a future for myself, and staying in the sport didn’t feel like the way to do that anymore”.

#### 3.2.6. Code 6: Struggles with Identity and Purpose Post-Sport

For five participants, the struggle to redefine their identity after leaving athletics was a significant challenge. Having dedicated much of their lives to their sport, they described feeling lost and uncertain about who they were without it.

“It was all I had ever done. Stepping away made me question everything about who I was and what I wanted”, shared P6. Similarly, P17 expressed, “I felt like I lost my purpose after leaving football. It was my escape, and without it, I didn’t know how to cope. It left a void I didn’t know how to fill”.

Some participants found that their identity was so intertwined with their sport that its absence left them feeling incomplete. P11 stated, “When I stopped playing, it was like a part of me was missing. I didn’t know how to describe myself to people anymore because being an athlete was who I was”.

For others, this identity shift prompted a search for new passions or goals. P8 explained, “Leaving the sport forced me to figure out who I was without it. It was hard, but it also made me realize there’s more to life than just being an athlete”.

The emotional weight of this transition was evident for P19, who reflected, “I still think about what my life would be like if I had stayed. But at the same time, I’m learning to embrace the fact that I’m more than just a player. It’s a work in progress”.

Even for participants who saw the decision as necessary, the adjustment remained challenging. P9 shared, “It felt like I had to start over. I was proud of what I had accomplished, but moving on meant figuring out what came next. That was scary”.

### 3.3. Theme 2: Barriers and Gaps in Institutional Mental Health Support

This theme highlights the perceptions of NCAA Division III student-athletes regarding the mental health resources available to them. Participants described systemic barriers such as stigma, insufficient access to resources and misaligned expectations between athletics and academics. These barriers were compounded by team cultures and inconsistent institutional follow-through on mental health initiatives. Some participants, however, highlighted examples of effective institutional support and proactive coaching. Table 10 summarizes the codes, participant involvement and transcript excerpts that contributed to this theme.

#### 3.3.1. Code 1: Lack of Mental Health Resources

Five participants described a lack of mental health resources as a major barrier, contributing to nineteen transcript excerpts. Counseling services, while available, were often described as inadequate, inaccessible or poorly suited to the needs of student-athletes. P8 shared, “There wasn’t anyone to talk to when I was struggling. The counseling center was overwhelmed, and they didn’t really understand athletes’ needs”. Similarly, P12 noted, “Our school had services, but they were the same ones everyone else used. Nothing specific for athletes, and it felt like mental health wasn’t really prioritized”.

Participants also mentioned logistical barriers to accessing these resources. P15 stated, “The counseling hours didn’t work with our practice schedule. By the time I was done with training, they were closed”. P3 added, “Even if I wanted help, it was hard to know where to go. There wasn’t a clear path to get support”.

#### 3.3.2. Code 2: Stigma for Male Athletes Seeking Support

Six participants highlighted stigma as a significant barrier to seeking mental health support, contributing eleven excerpts. This stigma was often associated with cultural norms around masculinity and athletic performance. P7 stated, “For guys, it’s hard to talk about mental health. You’re just expected to suck it up and keep going”. Similarly, P19 shared, “I think the stigma for male athletes is double—first for being male, then for being athletes. Everyone expects you to handle everything on your own”.

Some participants described the personal impact of this stigma. P4 explained, “I didn’t want anyone to think I was weak, so I just kept everything to myself”. P11 reflected, “You’re taught to push through pain, and that extends to mental health. It’s like, if you admit you’re struggling, you’re not tough enough to be here”.

#### 3.3.3. Code 3: Misaligned Expectations Between Sport and Academics

Four participants highlighted misaligned expectations between athletics and academics as a source of stress, contributing seventeen transcript excerpts. The dual demands of athletics and academics created a sense of being overwhelmed and unsupported. P10 remarked, “They say academics come first, but the athletic schedule doesn’t really allow for that. It felt like I was expected to give 100% to both, but that wasn’t realistic”.

P5 shared, “Balancing sports with school and everything else was honestly rough. I always felt like I was falling behind somewhere, whether it was my grades or just having a life outside of sports”. P16 elaborated on this tension, stating, “Practices and games took up so much time that it felt impossible to focus on school. I was constantly behind on assignments”.

#### 3.3.4. Code 4: Insufficient Institutional Follow-Through

Four participants described insufficient institutional follow-through on mental health initiatives, contributing sixteen transcript excerpts. Participants highlighted a disconnect between institutional messaging and actual support. P4 observed, “They talked a lot about mental health, but it felt like just talk. Nothing really changed or made a difference”.

P13 added, “There was a lot of emphasis on awareness, but no real action to back it up. It felt performative”. P20 noted, “They’d send emails about mental health resources, but when you actually needed help, there wasn’t anything available. It was frustrating”.

#### 3.3.5. Code 5: Team Cultures Promoting Poor Mental Health

Five participants discussed how team cultures negatively impacted their mental health, contributing eighteen excerpts. These cultures often emphasized performance and competitiveness at the expense of well-being. P6 explained, “It felt like all the coaches cared about was winning. If you weren’t performing, you didn’t matter”.

P17 described the team dynamic, stating, “Teammates were supportive to a point, but the culture was so competitive that it was hard to really connect or open up”. P11 noted, “The pressure to always be at your best didn’t leave room to talk about mental health. It was like you had to choose between being an athlete or being human”.

#### 3.3.6. Code 6: Case-by-Case Systemic Challenges

Four participants referenced unique systemic challenges, contributing sixteen transcript excerpts. P11 remarked, “I think it really depends on the school and the team. Some places really try to support you, but others just don’t care”. Similarly, P3 stated, “It wasn’t just the resources—it was the whole environment. It made everything harder”.

P9 shared an institutional example, “At my school, there was no real system for addressing mental health issues. If you had a problem, it was on you to figure it out”.

#### 3.3.7. Code 7: Comprehensive University Resources and Proactive Coaching

Despite the barriers, six participants highlighted examples of effective institutional support and proactive coaching, contributing ten excerpts. P9 explained, “My coach was amazing. She made sure we had the resources we needed and always emphasized that academics and mental health came first”.

P11 noted, “The university really made an effort to provide mental health resources. They organized workshops and created spaces where we could talk openly about our struggles”. P18 added, “We had a great support system. The coaches genuinely cared about us as people, not just athletes”.

### 3.4. Theme 3: External Pressures Influencing Decisions to Leave

This theme explores the external factors that influenced NCAA Division III student-athletes’ decisions to discontinue sports participation. Participants highlighted financial constraints, time and workload pressures, injuries, coaching dynamics and the prioritization of academics as well as long-term personal goals. These external pressures shaped their athletic experiences and often drove their decision to leave their sport. Table 11 summarizes the codes, participant involvement and transcript excerpts that contributed to this theme.

#### 3.4.1. Code 1: Financial Constraints

Six participants identified financial constraints as a significant factor influencing their decision to leave sports, contributing ten transcript excerpts. Financial burdens included the cost of tuition, equipment and the inability to work while maintaining athletic commitments. P9 explained, “I couldn’t afford to keep playing. Between tuition and all the extra expenses, it just didn’t make sense anymore”. Similarly, P15 shared, “Not being able to work during the season meant I couldn’t make enough money to cover my bills, so I had to step away”.

Some participants also noted that financial challenges exacerbated other pressures. P12 reflected, “Trying to manage school, sports, and finances was overwhelming. It was just too much to handle all at once”.

#### 3.4.2. Code 2: Time Constraints and Workload

Seven participants discussed how the time commitment and workload of being a student-athlete influenced their decision to leave, contributing twelve transcript excerpts. Balancing academics, athletics and personal responsibilities often became unmanageable. P10 remarked, “Practices, games, and schoolwork took up my entire day. I didn’t have time for anything else”.

P17 added, “I was barely making it to classes because of my athletic schedule. It felt like I was constantly running out of time to get everything done”. For some, the relentless schedule caused them to feel isolated. P7 explained, “I never had time to hang out with friends or just relax. It was like I was always on the clock”.

#### 3.4.3. Code 3: Physical Toll from Injuries or Health Challenges

Five participants highlighted the physical toll of injuries and health challenges as a critical factor in their decision to leave sports, contributing eighteen transcript excerpts. P8 shared, “I had a bad spinal injury in my freshman year. I tried to push through it, but it just got worse, and I had to stop”.

Others described how injuries affected their mental and physical well-being. P15 reflected, “The recovery process was long and exhausting. I couldn’t keep up with everything, and it felt like my body just couldn’t take it anymore”. P19 added, “After multiple concussions, I knew I couldn’t keep playing. It wasn’t worth risking my health”.

#### 3.4.4. Code 4: Coaching Staff Changes

Four participants discussed how changes in coaching staff affected their experiences, contributing seventeen transcript excerpts. P11 explained, “The new coaching staff didn’t care about us as individuals. It was all about winning, and it made me lose my passion for the sport”.

P4 described a similar experience, stating, “The coaches I committed to were great, but when they left, everything changed. The new coaches didn’t trust me, and it felt like I was starting over”. Some participants felt that these changes disrupted team dynamics. P20 reflected, “Every time a new coach came in, we had to adjust to a new system. It was exhausting and made it hard to stay motivated”.

#### 3.4.5. Code 5: Academic Priorities over Sports

Five participants emphasized the need to prioritize academics over athletics, contributing nineteen transcript excerpts. P13 stated, “I realized I couldn’t keep up with both my coursework and the demands of the sport. Something had to give”. P6 added, “I wanted to focus on my future career, and staying in the sport was holding me back academically”.

Others described how academic goals ultimately shaped their decisions. P3 noted, “I was struggling to maintain my grades, and it was clear that I needed to put school first”. P18 shared, “I came to college for an education, and I felt like the sport was getting in the way of that”.

#### 3.4.6. Code 6: Alignment with Personal Goals and Long-Term Plans

Five participants discussed how their personal goals and long-term plans influenced their decision to leave, contributing eighteen transcript excerpts. For some, leaving athletics allowed them to pursue other passions. P14 explained, “I wanted to explore other interests and opportunities outside of sports. It was time to move on”.

Others described how their priorities shifted over time. P9 reflected, “I loved playing, but I knew it wasn’t something I could do forever. I needed to focus on building a future for myself”. P19 added, “I realized that staying in the sport wasn’t aligning with my long-term goals. It was a hard decision, but it was the right one”.

### 3.5. Theme 4: Personal Reflections and Recommendations for Future Athletes

This theme reflects on participants’ personal journeys as NCAA Division III student-athletes, capturing their feelings of relief, regret, and growth after leaving sports. It also highlights their advice for balancing priorities, seeking support, and navigating the challenges of being a student-athlete. Table 12 summarizes the codes, participant involvement and transcript excerpts that contributed to this theme.

#### 3.5.1. Code 1: Seek and Use Available Support Systems

Seven participants emphasized the importance of seeking and utilizing available support systems, contributing twelve transcript excerpts. Many highlighted the value of leaning on teammates, coaches and institutional resources. P8 shared, “Find the support, find the help. It’s just a bonus to your life, but it isn’t your whole life”. Similarly, P9 stated, “Use the resources the school provides. Even if it feels like a small thing, talking to someone can really help”.

Participants also noted the need to seek advice from peers. P14 explained, “Lean on older teammates who have been through it before. They can give you insights and help you figure things out”. P3 added, “Teammates can be a huge source of strength. Don’t try to go through it alone when they’re there to help you”.

Some participants reflected on how institutional support systems could provide additional resources. P11 noted, “There’s counseling available if you need it, and it’s worth trying. I wish I had used it earlier”. P6 shared, “Coaches and advisors are there for a reason. Reach out to them—they want to help”.

#### 3.5.2. Code 2: Prioritize Personal Well-Being and Mental Health

Eight participants discussed the importance of prioritizing mental health and personal well-being, contributing fifteen transcript excerpts. Many reflected on how neglecting their mental health had long-term consequences. P7 stated, “Do what’s best for you and your mental health. It’s not worth sacrificing your happiness just to stay in the sport”.

P13 echoed this sentiment, “Your mental health has to come first. If you’re not taking care of yourself, everything else falls apart”. Others described how focusing on their well-being ultimately helped them grow. P11 reflected, “Stepping away was hard, but it allowed me to rebuild my mental health and focus on things that really mattered to me”.

Some participants described how ignoring mental health created additional strain. P12 explained, “I kept pushing myself even when I was struggling mentally, and it just made everything worse. I wish I had taken the time to step back earlier”. P15 shared, “Ignoring your mental health doesn’t make you stronger; it just makes things harder in the long run”.

#### 3.5.3. Code 3: Communicate Struggles Openly

Six participants highlighted the importance of open communication about struggles, contributing eleven transcript excerpts. P6 shared, “Don’t be afraid to speak up if you’re struggling. Talk to someone, whether it’s a teammate, a friend, or even a coach”.

Participants noted that keeping struggles hidden only made things worse. P10 explained, “If you don’t tell anyone what you’re going through, no one can help you. It’s better to be honest about what you need”. P15 added, “It’s okay to let people know you’re not okay. Everyone struggles, and talking about it can make a huge difference”.

Others described how sharing their struggles helped them find solutions. P17 shared, “When I finally opened up to my coach about what I was going through, they helped me figure out a way to manage things better”. P9 explained, “Talking about it didn’t fix everything, but it made me feel less alone and gave me some clarity”.

#### 3.5.4. Code 4: Balance Academics with Athletics

Five participants described the challenge of balancing academics with athletics and offered advice on managing both, contributing nineteen transcript excerpts. P9 explained, “You’re a student first. Don’t let your sport interfere with your performance in the classroom”.

P16 added, “Find a balance that works for you. It’s not easy, but if you stay organized, you can manage both school and sports”. Others encouraged prioritizing academics when necessary. P3 reflected, “There’s no shame in putting school first. At the end of the day, that’s why you’re here”.

Some participants noted that learning to balance these commitments took time. P14 explained, “It’s hard at first, but once you figure out how to manage your schedule, it gets easier”. P8 shared, “There were definitely times when school had to take priority over sports, and that’s okay”.

#### 3.5.5. Code 5: Reflect on Purpose and Goals

Six participants discussed the importance of reflecting on their purpose and long-term goals, contributing ten transcript excerpts. P19 shared, “Think about why you’re doing this and whether it aligns with what you want in life”. Similarly, P14 stated, “Take the time to figure out if staying in the sport is helping you achieve your goals or holding you back”.

For some, this reflection led to clarity about their priorities. P18 explained, “When I thought about my future, I realized that my sport wasn’t a part of it anymore. It helped me make peace with my decision to leave”. P12 added, “It’s important to think long-term. Your decisions now should align with where you want to be later”.

#### 3.5.6. Code 6: Encourage Perseverance but Normalize Stepping Away If Needed

Five participants emphasized the need to persevere through challenges while recognizing that stepping away is sometimes the best choice, contributing eighteen transcript excerpts. P12 stated, “It’s okay to step back if it’s what’s best for you. Don’t feel like you have to push through everything”.

P19 reflected, “Sometimes it’s better to walk away than to keep going when it’s hurting you. It’s not giving up; it’s choosing yourself”. Others noted the importance of resilience. P7 explained, “Stick with it if you can, but know that stepping away doesn’t mean you’ve failed. It just means you’re taking care of yourself”.

Several participants emphasized the need to balance perseverance with self-awareness. P17 explained, “Keep going if you still love it, but know when it’s time to let go. That’s the hardest but most important thing to figure out”.

#### 3.5.7. Code 7: Remember Why You Started and Reconnect with Motivation

Four participants encouraged future athletes to reconnect with their original motivation for participating in sports, contributing seventeen transcript excerpts. P6 shared, “Remember why you’re here. Think about what made you fall in love with the sport in the first place”.

P17 added, “When things get tough, remind yourself of your ‘why.’ It can help you push through the hard times”. For some, this perspective helped them rediscover their passion. P8 reflected, “I took a break, and it reminded me how much I loved the game. Sometimes you just need to step back to see the bigger picture”. P10 noted, “Reconnecting with why you started helps you stay grounded, even when things get hard”.

### 3.6. Theme 5: Sociology of Team Dynamics

This theme examines the social structures and interpersonal relationships within NCAA Division III athletic teams, emphasizing the impact of camaraderie, conflicts and team culture on athletes’ experiences. Participants reflected on the positive and negative dynamics within their teams and the role these interactions played in shaping their athletic journeys. Table 13 summarizes the codes, participant involvement and transcript excerpts that contributed to this theme.

#### 3.6.1. Code 1: Team Camaraderie

Six participants described how camaraderie within the team positively influenced their experiences, contributing ten transcript excerpts. P7 shared, “My teammates were like family. They were the reason I kept going, even when things got tough”.

Others reflected on the support they received from their peers. P14 stated, “Having a group of people who understood what you were going through made all the difference. We were all in it together”. P3 added, “The friendships I built on the team were some of the strongest relationships I’ve ever had”.

Camaraderie also contributed to athletes’ sense of belonging. P19 noted, “Being part of a team gave me a sense of purpose. It felt good to be part of something bigger than myself”.

#### 3.6.2. Code 2: Positive Relationship-Building Efforts

Five participants highlighted efforts by coaches and teammates to foster positive relationships, contributing eighteen transcript excerpts. P8 explained, “Our coach really emphasized team-building activities. We’d have dinners, group workouts, and even study sessions to make sure we were connected off the field”.

Participants also shared examples of teammates stepping up to support each other. P16 remarked, “Whenever someone was struggling, there was always someone willing to help. It created a really supportive environment”. P12 reflected, “Our team had this tradition of writing notes to each other before games. It was a small thing, but it showed we cared about each other”.

#### 3.6.3. Code 3: Mixed Teammate Support

Four participants described experiencing inconsistent levels of support from teammates, contributing seventeen transcript excerpts. P10 noted, “Some teammates were amazing—they’d have your back no matter what. Others were more focused on themselves and didn’t really care about the team dynamic”.

P17 added, “There were definitely cliques on the team, which made it hard to feel fully supported sometimes. You had to find your group and stick with them”. P6 reflected, “I felt supported most of the time, but there were moments when it felt like some people just didn’t care if you were struggling”.

#### 3.6.4. Code 4: Favoritism and Perceived Unfairness

Three participants discussed favoritism and perceived unfairness from coaches, contributing fifteen transcript excerpts. P11 shared, “It felt like the coaches had their favorites, and if you weren’t one of them, you didn’t get the same opportunities”.

P4 stated, “I worked just as hard as some of the starters, but I didn’t get the same recognition or chances to play. It was frustrating and made me question my place on the team”. P18 added, “Favoritism created a divide within the team. It was hard to feel like we were all in it together when some people were clearly treated differently”.

#### 3.6.5. Code 5: Disconnection or Exclusion

Four participants highlighted feelings of disconnection or exclusion within their teams, contributing eleven transcript excerpts. P9 explained, “I never really felt like I fit in with the team. It was hard to connect with people when they already had their groups”.

P20 reflected, “After my injury, I felt completely left out. It was like I didn’t exist anymore because I couldn’t contribute on the field”. P15 noted, “There were times when I felt excluded from team activities, and it made me question whether I really belonged there”.

#### 3.6.6. Code 6: Resolving Conflicts for Team Goals

Three participants described how conflicts within the team were addressed to achieve common goals, contributing fifteen transcript excerpts. P6 shared, “We didn’t always get along, but when it came down to it, we’d put our differences aside for the sake of the team”.

P12 added, “There was definitely drama at times, but we had a good system for resolving it. Our coach made sure we addressed issues head-on”. P17 reflected, “Even when there were disagreements, we always found a way to work together because we all wanted the team to succeed”.

#### 3.6.7. Code 7: Lack of Maintained Relationships

Four participants reflected on the difficulty of maintaining relationships with teammates and coaches after leaving the sport, contributing ten transcript excerpts. P14 explained, “Once I left the team, I lost touch with most of my teammates. It was like we were only connected because of the sport”.

P10 shared, “I still talk to a few people, but most of the relationships faded after I left. It’s hard to keep in touch when you’re not part of the team anymore”. P8 added, “I thought we’d all stay close, but life gets in the way, and it’s hard to keep those connections going”.

## 4. Discussion

Mental health challenges significantly impact NCAA Division III student-athlete retention, yet their experiences remain understudied [1,2,3]. This study sought to explore the lived experiences of former Division III athletes who voluntarily discontinued their sport, guided by three research questions introduced earlier: What mental health challenges did they face? How did they perceive institutional support? And what external pressures influenced their decision to step away? By returning to these questions, we aimed to highlight the personal and structural factors shaping these athletes’ decisions through the lens of transcendental phenomenology.

Five major themes were identified: emotional well-being, institutional support, external pressures, personal reflections and team dynamics. These findings illustrate how burnout, identity loss and systemic barriers intersect with cultural and institutional expectations, deepening our understanding of Division III sport discontinuation.

### 4.1. Theme 1: Emotional and Mental Health Challenges in Division III Athletics

The findings from this study highlight the profound emotional and psychological challenges faced by NCAA Division III student-athletes, particularly stress, burnout and identity loss. Many participants struggled with the overwhelming burden of balancing academic, athletic and personal responsibilities, which often led to emotional exhaustion. These results align with previous research showing that student-athletes are at a heightened risk of burnout due to the constant pressure to perform in multiple domains. According to a study by Gustafsson et al. (2017) [32], burnout among collegiate athletes is frequently linked to excessive training loads, academic stress and insufficient recovery time, all of which were reported by participants in this study. Furthermore, the relentless nature of their schedules left many athletes questioning their long-term well-being and future in their sport, leading to increased psychological distress.

A significant number of participants also reported experiencing symptoms of anxiety and depression, with some attributing these struggles to a perceived lack of institutional support. The NCAA’s 2021 Student-Athlete Well-Being Study found that mental health concerns, including mental exhaustion and anxiety, were highly prevalent among collegiate athletes, with nearly one-third of respondents reporting that they felt "mentally exhausted" on a frequent basis [33]. The current study’s findings support these concerns, as many participants described feelings of being overwhelmed, isolated and unsupported when facing mental health struggles. Additionally, the loss of enjoyment in their sport further compounded their distress, leading some to resent their athletic participation. Prior studies have suggested that the decline in intrinsic motivation among athletes is a key factor in burnout, particularly when external pressures from coaches and academic institutions override an athlete’s personal passion for the sport [34].

The emotional toll of leaving the sport was another significant theme, with many participants describing feelings of regret, relief and identity loss. Athletic identity is a crucial component of an athlete’s self-concept, and transitioning away from competitive sports can result in psychological distress if athletes struggle to redefine their sense of purpose [35]. Several participants in this study described the process of stepping away from their sport as akin to losing a fundamental part of themselves, an experience well-documented in previous research on athlete retirement and career transition. Studies suggest that athletes who lack alternative identities beyond their sport may face significant challenges in adjusting to post-athletic life, emphasizing the need for institutional support in helping athletes navigate this transition [36]. Overall, these findings underscore the necessity for universities and athletic programs to prioritize mental health resources, create robust support networks and foster an environment where student-athletes feel empowered to seek help without stigma.

### 4.2. Theme 2: Barriers and Gaps in Institutional Mental Health Support

The findings from this study highlight significant gaps in institutional mental health support for NCAA Division III student-athletes. Participants reported barriers such as inadequate access to counseling services, stigma—particularly among male athletes—misaligned expectations between athletics and academics and inconsistent institutional follow-through on mental health initiatives. These challenges align with prior research indicating that student-athletes face unique stressors that require specialized mental health resources [37]. However, many collegiate athletic programs, particularly at the Division III level, lack tailored services that address these needs [38]. The limited availability of athlete-specific mental health support often leaves student-athletes without the necessary tools to manage stress, anxiety and other psychological challenges effectively. This study’s findings reinforce existing concerns about the accessibility of mental health services and the logistical challenges that prevent student-athletes from utilizing them, such as conflicting practice schedules and the absence of sports-informed mental health professionals [39].

Stigma surrounding mental health support was another major barrier identified by participants, particularly among male athletes. Many expressed reluctance to seek help due to cultural norms emphasizing mental toughness and self-reliance, mirroring previous studies that suggest stigma is one of the most significant deterrents to help-seeking behaviors in athletes [40]. Collegiate sports environments often reinforce the idea that seeking psychological support is a sign of weakness, leading athletes to suppress their struggles rather than address them [39]. This stigma is especially pronounced among male athletes, who may experience additional societal pressure to conform to traditional notions of masculinity [41]. Addressing this issue requires a cultural shift within athletic programs, where mental health discussions are normalized, and seeking psychological support is encouraged rather than stigmatized. Educational initiatives targeted at both athletes and coaching staff can help dismantle harmful perceptions of mental health assistance and promote an environment where student-athletes feel comfortable accessing necessary support services [38].

Participants also highlighted misaligned expectations between academic and athletic responsibilities as a major source of stress, with many struggling to balance their coursework and training schedules. Although Division III athletics emphasize academic success, institutional structures often fail to accommodate the demands placed on student-athletes [37]. This misalignment can contribute to role conflict, increased stress, and burnout, ultimately affecting academic performance and overall well-being. Furthermore, inconsistent institutional follow-through on mental health initiatives was a recurring theme, as many participants felt that while universities promoted mental health awareness, tangible support remained insufficient [33]. The lack of systemic implementation of mental health policies suggests that institutional efforts may be performative rather than genuinely aimed at improving student-athlete well-being. However, despite these barriers, some participants reported positive experiences with proactive coaching and well-structured university support systems. These findings highlight the potential for athletic programs to foster healthier environments when mental health resources are integrated effectively. Expanding tailored mental health services, promoting a culture of openness, and ensuring institutional accountability in implementing support initiatives are crucial steps in improving the overall mental health landscape for Division III student-athletes.

### 4.3. Theme 3: External Pressures Influencing Decisions to Leave

The findings from this study highlight the significant role of external pressures in NCAA Division III student-athletes’ decisions to discontinue their sport. Financial constraints emerged as a major factor, as many athletes struggled with the costs associated with tuition, equipment and travel without the benefit of athletic scholarships. Unlike Division I and II programs, Division III institutions do not provide financial aid based on athletic ability, making it difficult for some athletes to sustain their participation [42]. The inability to work during the season further exacerbated financial burdens, forcing athletes to choose between their academic and athletic commitments. Prior research supports this, showing that financial considerations are a primary concern for student-athletes, especially in divisions where athletic scholarships are not available [43]. To improve retention, institutions should explore additional financial aid options, such as need-based assistance or stipends for student-athletes balancing multiple responsibilities.

Time constraints and workload were also critical factors, with participants describing an overwhelming schedule that left little room for rest, socialization or academic focus. The challenge of balancing coursework with demanding athletic schedules often resulted in stress and burnout, ultimately leading some athletes to reassess their participation. This aligns with previous studies suggesting that the mental and physical strains of juggling multiple responsibilities are significant predictors of athlete dropout [44]. Additionally, injuries played a major role in withdrawal decisions, as student-athletes who suffered long-term physical setbacks struggled with both the physical and emotional tolls of recovery. Injuries not only impact immediate performance but can also create long-term psychological stress, with some athletes experiencing anxiety, depression or a loss of athletic identity following injury [45]. Institutions must prioritize injury prevention, provide accessible rehabilitation services and offer psychological support to help athletes cope with the mental health effects of injuries.

Changes in coaching staff and academic priorities were also frequently cited as reasons for leaving their sport. Many participants described how shifts in coaching philosophy and interpersonal dynamics disrupted their motivation and commitment to their teams. Research indicates that coach–athlete relationships are central to athlete satisfaction and retention, with positive coaching behaviors enhancing motivation, while negative or inconsistent coaching can lead to disengagement [45]. Additionally, the emphasis on academics over athletics played a decisive role in withdrawal decisions, as many student-athletes ultimately prioritized their future career prospects. Division III athletics emphasizes the student-athlete experience rather than professional athletic development, making it more likely that athletes will step away when sports begin to interfere with their academic goals [33]. To better support student-athletes, institutions should work to align athletic and academic commitments more effectively, offering flexibility in practice schedules and promoting career development resources tailored to athletes transitioning out of competitive sports.

### 4.4. Theme 4: Personal Reflections and Recommendations for Future Athletes

The personal reflections of NCAA Division III student-athletes in this study highlight the importance of seeking and utilizing available support systems. Many participants acknowledged that reaching out to teammates, coaches and institutional resources played a crucial role in their overall well-being. This aligns with the NCAA’s Mental Health Best Practices, which emphasize the need for athletic departments to create accessible and supportive environments for student-athletes [46]. Prior studies have found that student-athletes who actively engage with available support services, including mental health counseling and academic advisors, report higher levels of resilience and overall well-being [47]. However, the reluctance to seek support due to stigma or lack of awareness remains a persistent challenge, reinforcing the need for increased education on the benefits of utilizing these resources.

Prioritizing mental health and personal well-being emerged as another significant theme, as participants described the consequences of neglecting their mental health. The NCAA has identified mental health as a top priority, advocating for increased awareness and proactive interventions [48]. Research indicates that student-athletes often experience heightened stress, anxiety, and burnout due to the demands of balancing academics and athletics, making it critical for institutions to foster environments where mental health support is normalized [46]. Furthermore, open communication about struggles was highlighted as an essential strategy in mitigating mental health challenges. When athletes feel comfortable discussing their difficulties with coaches, teammates or mental health professionals they are more likely to receive timely assistance and develop effective coping strategies [47]. Encouraging this level of openness within athletic programs can reduce stigma and foster a culture of psychological safety for student-athletes.

Balancing academics with athletics was a recurring concern among participants, as many struggled with the time demands of both commitments. Division III athletics explicitly prioritizes academic achievement and emphasizes shorter playing and practice seasons to allow student-athletes to focus on their coursework [33]. However, despite these structural safeguards, many athletes still experience significant challenges in time management, highlighting the need for additional institutional support in developing academic–athletic balance strategies. Finally, reflecting on personal goals and motivations played a key role in how participants navigated their experiences. Many athletes recognized the value of aligning their athletic participation with their long-term aspirations, ensuring that their sport enhanced rather than hindered their overall development [33]. Encouraging student-athletes to regularly reassess their goals and maintain a broader perspective on their future can help them make informed decisions about their participation in athletics.

### 4.5. Theme 5: Sociology of Team Dynamics

The findings from this study emphasize the critical role of team camaraderie and interpersonal relationships in shaping NCAA Division III student-athletes’ experiences. Many participants described their teammates as a source of emotional and motivational support, reinforcing existing research that highlights the importance of social belonging in collegiate sports. The NCAA Division III 2024–25 Facts and Figures report found that 78% of student-athletes agreed that their coaches and teammates created an inclusive environment, suggesting that strong team bonds contribute to overall satisfaction and retention in sports [48]. Additionally, research has shown that athletes who feel connected to their teammates are more likely to experience higher levels of engagement and lower rates of burnout [32,34]. Given these findings, fostering inclusive and cohesive team cultures should be a primary focus for coaches and athletic departments.

However, while many participants reported positive team relationships, others described challenges such as favoritism, cliques and exclusion, which negatively impacted their sense of belonging. Favoritism from coaches, perceived unfair treatment and feeling disconnected from teammates have been shown to decrease motivation and contribute to sport discontinuation [49]. Research suggests that team culture plays a crucial role in mitigating these issues, with effective coaching and leadership fostering environments that prioritize equity and collective success [50]. The presence of social subgroups or cliques within teams can create divisions, reinforcing disparities in opportunities and support. To address this, coaches should actively promote open communication, fair playing opportunities and structured team-building activities to ensure all athletes feel valued and included.

Maintaining relationships after leaving collegiate athletics also emerged as a significant challenge for former athletes, with many expressing that their friendships and team connections faded over time. This aligns with prior research showing that athletic identity is strongly tied to an athlete’s social environment, making the transition out of sports particularly difficult [49]. The lack of sustained relationships post-college suggests that institutions and athletic departments could implement alumni networking opportunities or mentorship programs to help former athletes maintain their connections and continue benefiting from the support of their athletic communities. Ultimately, ensuring a positive and inclusive team culture while in college can lead to lasting social benefits that extend beyond an athlete’s competitive years.

Taken together, these findings align with and expand upon the issues raised in the Introduction: limited institutional mental health resources, high performance demands and insufficient attention to the experiences of Division III athletes. By centering participants’ lived experiences, this study not only validates previously recognized challenges but also introduces new insights into how internal struggles and external constraints interact over time. The themes identified reflect more than just common experiences, they represent systemic patterns that merit structural attention from athletic departments, university administrations and governing bodies such as the NCAA.

Future interventions should be rooted in these experiential accounts to ensure that support systems are attuned to the specific needs and contexts of Division III student-athletes.

### 4.6. Study Limitations

While this study provides important insights, there are some limitations. With 21 participants, the sample size allowed for the rich, in-depth exploration of lived experiences, though findings should be interpreted as context-specific rather than broadly generalizable. As with most phenomenological research, all data were self-reported. However, self-reporting can be interpreted as a methodological strength that centers participants’ voices and perspectives. This study includes only athletes who chose to discontinue their sport, which limits insights into those who persisted despite experiencing similar challenges. It also does not account for variations in mental health resources across institutions, which may influence how athletes experience and navigate psychological stressors. Future research could incorporate longitudinal or mixed-method approaches to explore how Division III athletes respond to mental health interventions over time and across different institutional contexts.

## 5. Conclusions

This study shows how mental health struggles, lack of support and outside pressures push NCAA Division III athletes to quit their sports. Stress, burnout, anxiety and identity loss were major factors, worsened by limited mental health resources and stigma around seeking help. Financial issues, time management, injuries and coaching changes also played a role. The findings highlight the need for better mental health support, stronger team cultures and efforts to reduce stigma. Schools should offer more financial aid and flexible scheduling to ease outside pressures. Addressing these issues can improve athlete well-being and retention. Future research should focus on strengthening mental health support in college athletics.

## Figures and Tables

**Table 1:** Interview questions with probes.

Question	Question	Probes	Purpose
1	Can you tell me about your experience as a student-athlete in NCAA Division III?	What motivated you to play at this level? What did you enjoy most about being a student-athlete?	Icebreaker; provides context for understanding overall experience.
2	At what point did you start thinking about leaving your sport?	Was there a specific event or moment that led to this decision? How long did you consider it before stepping away?	Addresses timeline and thought process, and identifies factors influencing decision to quit.
3	What were the main factors that led to your decision to leave?	Were these factors related to physical, mental, or external pressures? Were any related to the balance between academics and athletics?	Identifies barriers and challenges influencing decision to quit.
4	How did your mental health play a role in your decision to step away from athletics?	Were there any specific mental health struggles that contributed to your decision? Did you feel supported in addressing these issues?	Explores mental health’s influence on athlete retention.
5	Can you describe the support system available to you as a student-athlete in terms of mental health and well-being?	Did your school or team provide access to counseling or mental health services? Did you feel comfortable seeking help?	Explores resources available to student-athletes.
6	How did you feel about balancing your athletic responsibilities with your academic or personal life?	Did this balance create stress? How did you manage the demands of both?	Highlights stressors beyond athletics contributing to mental health challenges.
7	What, if any, challenges did you face in maintaining relationships with coaches or teammates?	Did you feel supported by your coaches or teammates when you were struggling? Were there any difficult conversations with your coach about leaving?	Identifies interpersonal challenges as a potential barrier to retention.
8	How did you feel after making the decision to leave your sport?	Did you experience any relief, regret, or other emotional responses? How did this decision impact your overall well-being?	Explores the emotional consequences of quitting and mental health effects post-decision.
9	If you had received more or different types of support, do you think you would have made a different decision?	What type of support would have been helpful? What role could mental health resources have played in your decision?	Explores how different interventions or support systems could affect retention.
10	What advice would you give to current DIII athletes who might be struggling with the same issues you faced?	What would you suggest they do in terms of seeking help? What role do you think mental health awareness should play in college athletics?	Gathers insights into how future athletes can navigate similar challenges.
11	Reflecting on your experience, do you think there are systemic issues in Division III athletics that contribute to mental health challenges or athlete retention?	Are there institutional changes you would suggest? How could DIII programs better support their athletes?	Identifies systemic factors contributing to mental health barriers and retention issues.
12	Is there anything else you would like to share about your experience that we haven’t covered?	Anything regarding mental health, coaching, team dynamics, or school resources?	Allows participants to offer additional insights relevant to the research.

**Table 2:** Sports played by participants.

Sport	Number of Participants
Softball	3
Tennis	1
Soccer	2
Women’s soccer	1
Field hockey	1
Football	6
Lacrosse	2
Baseball	2
Track and field	1
Hockey	1
Swimming	1

**Table 3:** Participant sex distribution.

Sex	Number of Participants	Percentage
Male	13	62%
Female	8	38%

**Table 4:** Age at discontinuation.

Age	Number of Participants
18 years	2
19 years	4
20 years	7
21 years	5
22 years	2
23 years	1

**Table 5:** Academic majors of participants.

Academic Major	Number of Participants
Psychology and sports medicine	1
Human services and rehabilitation	1
Public health	2
Applied exercise science	2
Exercise science	3
Human resources	1
Undecided	1
Management	1
Criminology	1
Astrophysics	1
Biology	2
Athletic training	1
Finance	1
Economics	1
Business	2
Accounting	1

**Table 6:** Timing of sports discontinuation.

Timing of Discontinuation	Number of Mentions
First year of college	3
Second year of college	4
Third year of college	5
Fourth year of college	3
After an injury	3
Mid-season withdrawal	3

**Table 7:** Reasons for sports discontinuation.

Reason for Discontinuation	Participants
Burnout from sports participation	3
Mental health struggles (stress, anxiety and depression)	4
Financial concerns impacting well-being	2
Short-term commitment (e.g., “about 6–8 months”)	1
Personal dissatisfaction with collegiate sports	3
Lack of team support	2
Overwhelming academic and athletic demands	3
Social pressure	2
Injury-related mental health struggles	1

**Table 8:** Themes.

Theme	Description
1. Emotional and Mental Health Challenges in Division III Athletics	Captures the emotional toll faced by Division III athletes, including stress, burnout and identity loss, which impacted their well-being and participation.
2. Barriers and Gaps in Institutional Mental Health Support	Highlights athletes’ perceptions of mental health resources, emphasizing systemic barriers like stigma, lack of access and misaligned expectations, alongside examples of effective support.
3. External Pressures Influencing Decisions to Leave	Explores external factors such as financial constraints, coaching dynamics and academic priorities that influenced athletes’ decisions to leave sports.
4. Personal Reflections and Recommendations for Future Athletes	Reflects on athletes’ personal journeys, capturing feelings of relief or regret, and offers advice for balancing priorities and seeking support.
5. Sociology of Team Dynamics	Examines the social structures and interpersonal relationships within teams, highlighting the impact of camaraderie, conflicts and team culture on athletes’ experiences.

**Table 9:** Theme 1: Emotional and Mental Health Challenges: codes.

Codes
1. Stress and burnout
2. Loss of enjoyment and purpose
3. Depression and anxiety
4. Emotional toll from leaving the sport
5. Burnout and reflection on personal goals
6. Struggles with identity and purpose post-sport

**Table 10:** Theme 2: Barriers and Gaps in Institutional Mental Health Support: codes.

Codes
1. Lack of mental health resources
2. Stigma for male athletes seeking support
3. Misaligned expectations (sport/academics)
4. Insufficient institutional follow-through
5. Team cultures promoting poor mental health
6. Case-by-case systemic challenges
7. Comprehensive university resources

**Table 11:** Theme 3: External Pressures Influencing Decisions to Leave: codes.

Codes
1. Financial constraints
2. Time constraints and workload
3. Physical toll from injuries or health challenges
4. Coaching staff changes
5. Academic priorities over sports
6. Alignment with personal goals and long-term plans

**Table 12:** Theme 4: Personal Reflections and Recommendations for Future Athletes: codes.

Codes
1. Seek and use available support systems
2. Prioritize personal well-being and mental health
3. Communicate struggles openly
4. Balance academics with athletics
5. Reflect on purpose and goals
6. Encourage perseverance but normalize stepping away if needed
7. Remember why you started and reconnect with motivation

**Table 13:** Theme 5: Sociology of Team Dynamics: codes.

Codes
1. Team camaraderie
2. Positive relationship-building efforts
3. Mixed teammate support
4. Favoritism and perceived unfairness
5. Disconnection or exclusion
6. Resolving conflicts for team goals
7. Lack of maintained relationships

## Data Availability

The data that support the findings of this study are available from the corresponding author upon reasonable request.
